# Dietary Supplementation of Tannins: Effect on Growth Performance, Serum Antioxidant Capacity, and Immunoglobins of Weaned Piglets—A Systematic Review with Meta-Analysis

**DOI:** 10.3390/antiox13020236

**Published:** 2024-02-15

**Authors:** Emmanuel Nuamah, Junior Isaac Celestin Poaty Ditengou, Fabrice Hirwa, Inhyeok Cheon, Byungho Chae, Nag-Jin Choi

**Affiliations:** Department of Animal Science, Jeonbuk National University, Jeonju 54896, Republic of Korea; celestinisaacjunior10@jbnu.ac.kr (J.I.C.P.D.); fabugih@jbnu.ac.kr (F.H.); cheon6664@naver.com (I.C.); byungho721@gmail.com (B.C.)

**Keywords:** tannins, weaned piglets, antibiotics, antioxidants, immunity, performance, early-life nutrition, meta-analysis

## Abstract

**Simple Summary:**

Tannins, which are plant bioactive compounds, have the potential to improve productive performance, reduce oxidative stress, and enhance the immune indices of weaned piglets. Hence, the objective of this study was to evaluate the effect of dietary supplementation of tannins on weaned piglets’ growth performance, serum antioxidant capacity, and serum immune status using a meta-analysis approach. Supplementation of tannins enhanced performance by reducing the feed conversion ratio while increasing the final body weight of piglets. It also increased the blood serum’s immune indices and antioxidant capacity. The results revealed that tannins can be explored in the weaning phase to impact production efficiency and health in the late growth phase.

**Abstract:**

In recent years, the swine industry has witnessed the withdrawal of antibiotics and continuous regulation of zinc and copper oxides in the early-life nutrition of piglets. Due to this development, alternative additives from plant sources have been extensively explored. Therefore, this study’s objective was to evaluate the effect of dietary supplementation with tannins on weaned piglets’ growth performance, serum antioxidant capacity, and serum immune status using a systematic review and meta-analysis approach. A total of 16 studies with parameters of interest were deemed eligible after a two-step screening process following a comprehensive literature search in the scientific databases of Web of Science, Scopus, ScienceDirect, PubMed, and Google Scholar. The inclusion criteria were mainly (1) studies involving basal diet supplemented with tannins and (2) studies with the quantification of tannin doses, while the exclusion criteria were (1) studies with pre- and post-weaning pigs and (2) challenged studies. Applying the random-effects models, Hedges’ g effect size of supplementation with tannins was calculated using R software to determine the standardized mean difference (SMD) at a 95% confidence interval. Sub-group analysis and meta-regression further explored heterogeneity (P*_SMD_* < 0.05, *I*^2^ > 50%, *n* ≥ 10). Supplementation with tannins reduced the feed conversion ratio (*p* < 0.01) but increased the final body weight (*p* < 0.01) of weaned piglets. Chestnut and grape seed proanthocyanidin tannin sources yielded higher effects on growth performance. In addition, meta-regression models indicated that tannin dosage and supplementation duration were directly associated with tannins’ effectiveness on productive performance. In the serum, the concentration of glutathione peroxidase, superoxide dismutase, and total antioxidant capacity were elevated (*p* < 0.01) in response to tannin supplementation, whereas malondialdehydes was reduced (*p* < 0.01). Likewise, increased immunoglobin M and G levels (*p* < 0.01) were detected. In conclusion, dietary supplementation with tannins, particularly with chestnut and grape seed proanthocyanidins, increases the productivity of weaned piglets. At the same time, it is a possible nutritional strategy to mitigate oxidative stress and stimulate gut health. Thus, supplementing chestnut and grape seed proanthocyanidin tannins in the early phase of swine production could be used to alleviate the incidence of diarrhea.

## 1. Introduction

Weaning, a common and obligatory husbandry practice, is recognized to be one of the most critical phases in the modern swine industry [1]. Recently, piglets have been weaned at 19–25 days to increase sow reproductive efficiency, thus improving annual productivity [2]. However, this early weaning, which is linked to immature digestive and immune organs [3], injury of the gastrointestinal barrier, and a decline in metabolism [4], poses substantial physiological, environmental, and social stressors for piglets, including abrupt separation from their mothers, exposure to unfamiliar piglets, establishment of a new social hierarchy, different housing conditions, and changes in feed sources [2,5]. During this phase, piglets are exposed to the risk of severe diarrhea, digestion, intestinal absorption, growth retardation, and even death, which usually cause enormous economic losses to the swine industry [6,7].

Hence, in diets for weaned piglets, a variety of antibiotics and chemical growth promoters, including zinc oxide (ZnO) and copper oxide (CuO), have been explored to improve piglets’ health [8]. Yet, the indiscriminate use of antibiotics has increased bacteria resistant to their effects, representing a significant threat to the health of animals and humans [9]. Likewise, zinc and copper oxide in pharmacological doses [10] are being phased out by the European Commission as a veterinary instrument in the entire union; their use is currently regulated in other countries due to their potency as heavy metal contaminants in the environment and the issue of resistance to certain bacteria [11,12,13]. Thus, prohibiting antibiotics, zinc, and copper oxide in piglet diets poses a significant challenge for the swine industry. This situation has spawned researchers’ interest in exploring and developing new natural alternatives possessing efficient properties to maintain the gut health of piglets during weaning, thereby further supporting animal performance [14,15]. Among these natural alternatives, tannins, a secondary plant metabolite, hold immense potential.

Tannins, an astringent group of polyphenolic compounds [16] of high molecular weight [17], mainly existing in a wide variety of plants, are classified into hydrolyzable tannins (HTs) and condensed tannins (CTs) based on their chemical structure [18,19]. Condensed tannins are polymers of flavin-3-ols, flavin-4-diols, or related flavanol residues linked via carbon–carbon bonds [20]. Hydrolyzable tannins, conversely, are heterogeneous groups of natural polyphenolic water-soluble compounds widely found in vegetable feedstuffs and can be extracted from the wood of trees [21]. The unique structures and mechanisms of tannins provide beneficial effects from their antimicrobial, antioxidant, radical scavenging, antidiarrhea, anticancerogenic, and anti-inflammatory activities in weaned pigs [9,22]. Numerous studies have highlighted tannins’ benefits and challenges in monogastric animals’ health and productivity [23]. Recent studies have demonstrated that basal diet supplemented with tannins could improve health status and animal performance and positively affect small intestine morphometric traits in monogastric animals [24,25,26]. However, tannins, known for their anti-nutritional properties, also negatively affect growth performance [22]. In fact, tannins decrease feed palatability and ingestion [27] and can reduce the digestibility of dietary protein [28] due to their insoluble complex forming ability with both protein [29] and digestive enzymes [30].

Remarkably, in weaned piglets, heterogeneous results have been reported for the effects of dietary tannins to enhance growth performance and antioxidant status in the blood, modulate intestinal microbiota, and decrease the incidence of diarrhea during the post-weaning period [17,22,24,31,32,33,34,35,36,37,38]. According to Huang et al. [34], the chemical characteristics of tannins could be related to these inconsistent results, which can compromise the palatability, digestibility, and protein use of feed and thus render the results obtained to date heterogeneous and inconclusive, probably as a consequence of discrepancy among studies regarding the feeding conditions, age of the piglets, type of tannins (HTs or CTs), source of tannins, dosage of tannins, and duration of tannin supplementation [23,24,39]. Therefore, it has become imperative to investigate and control the source of heterogeneity as a critical attribute in developing tannin-containing products to enhance the growth performance, serum antioxidant capacity, and serum health indices of weaned piglets.

Few studies presented in the literature have reviewed tannin supplementation in terms of improving livestock’s productive performance and health status [23,40,41,42,43,44,45,46,47,48], with the majority adopting a narrative approach. However, none of these reviews focused on weaned piglets using a meta-analytic method. Meta-analysis (MA), according to Higgins and Green [45], is a statistical method used to combine results from the relevant studies, and the resultant larger sample size provides more precise reliability of the estimates than any treatment effect. Additionally, the heterogeneity sources among diverse related studies can be explored using MA, which helps obtain additional information about the variability of the observed outcomes in response to a specific treatment [49]. Although the MA use in animal nutritional studies is gaining momentum, its utilization in tannin supplementation in weaned piglets’ is scarce. Hence, we hypothesized that tannin supplementation in the basal diet would positively modify the productive performance, serum antioxidant status, and immune indices of piglets. Therefore, this meta-analysis aimed to evaluate the effect of dietary supplementation with tannins on weaned piglets’ growth performance, serum antioxidant capacity, and serum immune status. We further explored the heterogeneity of the effect size of the outcomes by subgroup analysis and meta-regression analysis.

## 2. Materials and Methods

### 2.1. Literature Search and Study Selection

**Figure 1:**
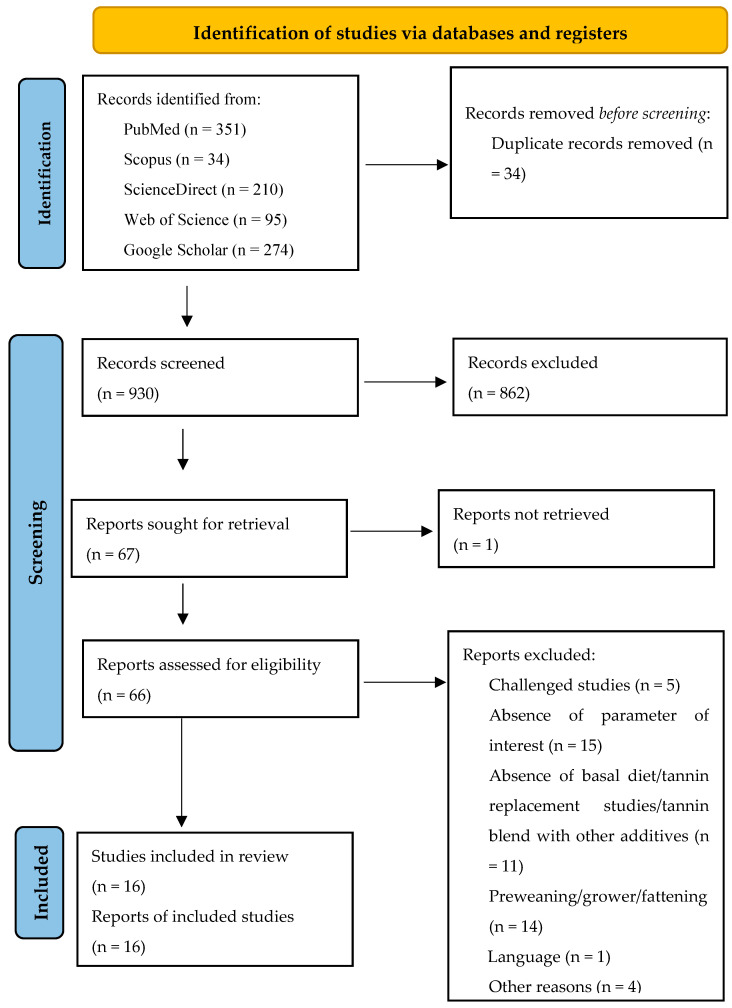
Systematic literature search and selection process. The PRISMA diagram details the applied search and selection process of this study (PRISMA checklist is in Supplementary Materials).

This meta-analysis (registration number: INPLASY202410093) followed the Preferred Reporting Items for Systematic Reviews and Meta-Analysis (PRISMA) updated guidelines [50] for identifying, selecting, choosing, and including information, as shown in Figure 1.

To identify studies that evaluated the effects of tannin supplementation on productive performance, antioxidant status, and immune indices of weaned piglets, a comprehensive literature search in the scientific databases Web of Science (accessed on 20 October 2023), Scopus (accessed on 20 October 2023), ScienceDirect (accessed on 20 October 2023), PubMed (accessed on 20 October 2023), and Google Scholar (accessed on 20 October 2023) was carried out. The search was limited to the results of papers published between 2010 and 2023. In all the databases, the keywords “tannin”, “condensed tannins”, “hydrolyzable tannin”, “weaned pig*”, “growth”, “antioxidant status”, and “immune indices” were used.

### 2.2. Inclusion and Exclusion Criteria

Search results from the five databases were pooled in Zotero (Version 6.0.30), and then duplicate publications were removed. The remaining records were independently screened by two reviewers through a two-step process, as previously described by other authors [45,51]. First, a screening was performed using the title and abstract, excluding review papers, stimulated studies (in vitro), and studies not including weaned pigs/piglets. Papers that passed the title and abstract screening were assessed for eligibility in the second step based on the inclusion and exclusion criteria of the meta-analysis. Inclusion criteria: (1) peer-reviewed journal article published in English, (2) studies involving basal diet supplemented with tannins, (3) studies on crossbred weaned pigs, (4) studies with a randomized allotment of weaned pigs, (5) studies with a quantified dose of tannins, (6) studies that reported the means of the control and experimental group with variability measures (standard deviation or standard error of mean) and sample size, and (7) studies that reported the parameters of interest. The exclusion criteria included (1) challenged studies, (2) studies with pre- and post-weaning pigs, (3) studies with tannins fed as a replacement ingredient in the diet of weaned pigs, and (4) studies with tannins combined/blended with probiotics/prebiotics/organic acids or other additives.

### 2.3. Data Extraction

Two main categories of data were independently extracted by two reviewers from the eligible studies. The study characteristics of author, year, country, breed/strain, age at weaning, supplementation duration, tannin type, source, number of piglets, and average initial weight were extracted from each study. The response variables came from three categories. The growth performance category included average daily feed intake (ADFI), average daily gain (ADG), final body weight (FBW), and feed conversion ratio (FCR). The antioxidant parameters included glutathione peroxidase (GSH-Px), superoxide dismutase (SOD), catalase (CAT), malondialdehydes (MDA), and total antioxidant capacity (T-AOC). The third category, immune indices, included immunoglobulin A (IgA), immunoglobin G (IgG), and immunoglobin M (IgM). The mean, standard deviation, or standard error of all outcomes corresponding to tannins and control groups were extracted from each study. Each treatment was considered a separate trial for studies including more than one treatment. The data extracted from eligible studies were compiled using an electronic form created in Microsoft Excel (Microsoft Corp., Redmond, WA, USA).

### 2.4. Study Quality Assessment

Two researchers independently assessed the study quality using the Cochrane Collaboration’s Systematic Review Center for Laboratory Animal Experimentation’s (SYRCLE) Risk of Bias (RoB) tool for animal studies [52]. The assessment items included random sequence generation (selection bias), baseline characteristics (selection bias), allocation concealment (selection bias), random housing (performance bias), blinding of participants and personnel (performance bias), random outcome assessment (detection bias), incomplete outcome data (attrition bias), selective reporting (reporting bias), and other biases. Discussions with a third researcher settled the disagreements in assessment.

### 2.5. Statistical Analysis

#### 2.5.1. Meta-Analysis

All the statistical analyses were performed using R software (version 4.3.1, The R Foundation for Statistical Computing, 16 June 2023 ucrt) using the “meta” and “metafor” packages. The means of the experimental units (control and treatment) were registered as continuous result data. The response variables were analyzed through the standardized mean difference (SMD), also called the effect size (ES), in which the difference between the means of the experiment and the control was standardized using the standard deviation (SD) of the groups with and without tannins. The random-effects model was used to estimate the effect size, with a 95% confidence interval (CI) and the statistical significance for each trait, since it is more conservative than the fixed-effects model [53,54]. SMD values of <0.2, 0.2 < SMD < 0.7, and >0.7 indicated small, moderate, and high effects, respectively [55,56]. Eleven meta-analyses (global studies) were run separately for each response variable studied, growth performance (ADFI, ADG, FBW, and FCR), antioxidant parameters (GSH-Px, SOD, CAT, MDA, and T-AOC), and immune indices (IgA, IgG, and IgM). The effect sizes of each experimental unit comparing tannin impacts were calculated for each outcome variable with Hedges’ g. A *p*-value of SMD less than 0.05 was considered statistically significant.

#### 2.5.2. Heterogeneity Assessment

The effect size heterogeneity was measured using Cochran’s Q test and the *I*^2^ (percentage of variation) statistics. Heterogeneity between-study variability was assessed using values ranging from 0 to 100% (*I*^2^ < 25% = low heterogeneity, 25% ≤ *I*^2^ ≤ 50% = moderate heterogeneity, 50% ≤ *I*^2^ < 75% = high heterogeneity, 75% ≤ *I*^2^ ≤ 100% = very high heterogeneity) [49]. Sub-group analysis and meta-regression were necessary to determine the sources of heterogeneity further when the studies had a substantial heterogeneity (*I*^2^ > 50%) [49].

#### 2.5.3. Meta-ANOVA and Meta-Regression

Meta-ANOVA (sub-group analysis) tests were conducted to compare the effects of the tannin sources (chestnut, quebracho, carob pods, gallnut microencapsulated tannic acid, gallnut tannic acid, grape seed proanthocyanidins, and chestnut and quebracho blend). Meta-regression analysis was conducted using effect sizes (SMD) for each outcome (P*_SMD_* < 0.05, *I*^2^ > 50%, n ≥ 10) as the dependent variable to examine heterogeneity sources of meta-analysis with tannin dosage (mg/kg), supplementation duration (days), and piglets’ age at weaning (days) as a covariate.

#### 2.5.4. Publication Bias

Publication bias was analyzed to confirm the study results’ validity and assess the risk of bias in individual studies. The funnel plots were drawn to visualize the bias, and Egger’s linear test was performed to evaluate the publication bias accurately with numerical data [57]. Tests to assess publication bias can be achieved when the variable to be considered is at least ten studies and when significant heterogeneity (Q) is detected with *p* ≤ 0.05 because it may lead to false-positive claims [58]. Consequently, funnel plots and Egger’s test were only performed for variables that met the criteria above. In cases where statistical evidence of publication bias was found, Duval and Tweedie’s “trim-and-fill” method was used to estimate the number of possible missing observations [59].

## 3. Results

### 3.1. Dataset and Study Characteristics

The strategy, process, and results of our literature search are shown in Figure 1. A total of 964 articles were identified from PubMed, Scopus, ScienceDirect, Web of Science, and Google Scholar for screening. In our screening process, duplicates and ineligible papers were removed, with a final total of 16 papers registered for data extraction and meta-analysis for our review. The 16 papers were divided into 31 experiments because some studies had several tannin supplementation levels (treatments). An experiment was defined as the control diet associated with one tannin supplementation level. The summary characteristics of the primary studies (16 papers) included in the meta-analysis are shown in Table 1.

The studies included in this meta-analysis were conducted in seven different countries, predominantly in China (50%), Greece (12.5%), and Germany (12.5%). The average weaned age of piglets (crossbreds) was 25 days, with a minimum of 21 days and a maximum of 35 days, while the experimental duration varied between 14 and 55 days. In addition, the average weight of the piglets ranged between 5.99 and 10.70 Kg. Regarding the type of tannins, most of the studies in this meta-analysis supplemented condensed tannins, which constituted 50%. In contrast, hydrolyzable tannins comprised 43.75%, and a blend of condensed and hydrolyzable tannins made up 6.25%. Regarding the source of tannin supplementation, 18.75% of the studies used chestnut and grape seed proanthocyanidins individually, followed by gallnut tannin acids and GMTA, which comprised 12.50% singly. In addition, grape pomace constituted 12.50%, whereas grape extract, carob pods, a blend (Ch/Qu), and quebracho, each comprising 6.25%. The included studies supplemented the tannins in a dosage ranging from 40 to 12,500 mg/kg.

### 3.2. Assessment of Risk of Bias

**Figure 2:**
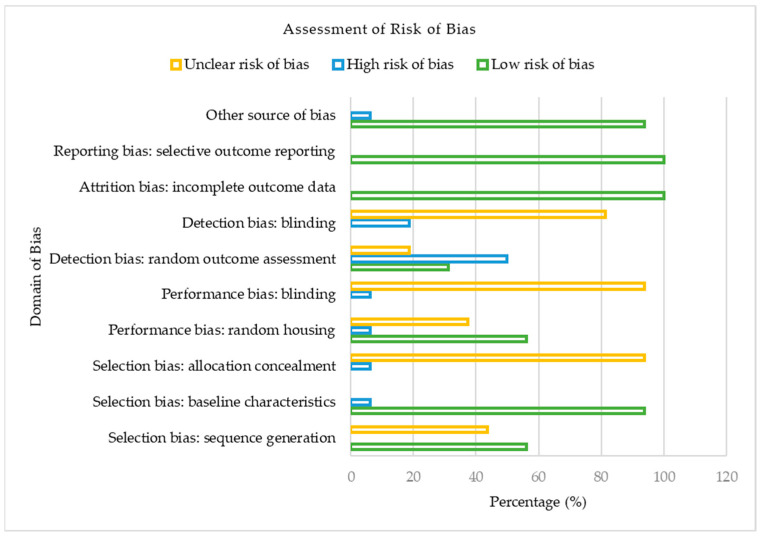
A bar chart of the risk of bias classification of the included studies based on the Cochrane Collaboration’s SYRCLE Risk of Bias tool for animal studies.

Figure 2 presents the risk of bias classification for the studies included in our meta-analysis based on the Cochrane Collaboration’s SYRCLE Risk of Bias tool for animal studies.

Regarding selection bias, out of the 16 included studies, 9 (56.25%) reported a low risk of bias, whereas 43.75% were assessed as having an unclear risk of bias for the sequence generation domain. Likewise, the majority (93.75%) of the studies were judged as having low risk of bias, though 6.25% had a high risk of bias based on their baseline characteristics. In contrast, for allocation concealment, the majority (93.75%) of the included studies showed an unclear risk of bias, with just 6.25% constituting a high risk of bias. Considering the performance domain of the risk of bias assessment, 56.25%, 37.50%, and 6.25% of the studies presented a low, unclear, and high risk of bias concerning random housing. With no low risk of bias reported concerning the blinding of caregivers, the included studies showed a 93.75% unclear risk of bias, whereas 6.25% were judged as having a high risk of bias. Regarding the detection bias of the included studies, random outcome assessment was adjudged as the domain with the utmost proportion of high risk of bias (50.00%) in our meta-analysis. On the other hand, blinding of outcome assessors, another domain of detection bias, showed 18.75% and 81.25% low and unclear bias, respectively. For both attrition and reporting bias, the studies of our meta-analysis were all judged to present a 100% low risk of bias. In our included studies, 93.75% and 6.25%, respectively, were deemed to have a low and unclear risk of bias for other sources of bias. In summary, of the 16 eligible studies in our meta-analysis, approximately 53% had a low risk of bias, 37% had an unclear risk of bias, and 10% constituted a high risk of bias.

### 3.3. Meta-Analysis

#### 3.3.1. Growth Performance

**Figure 3:**
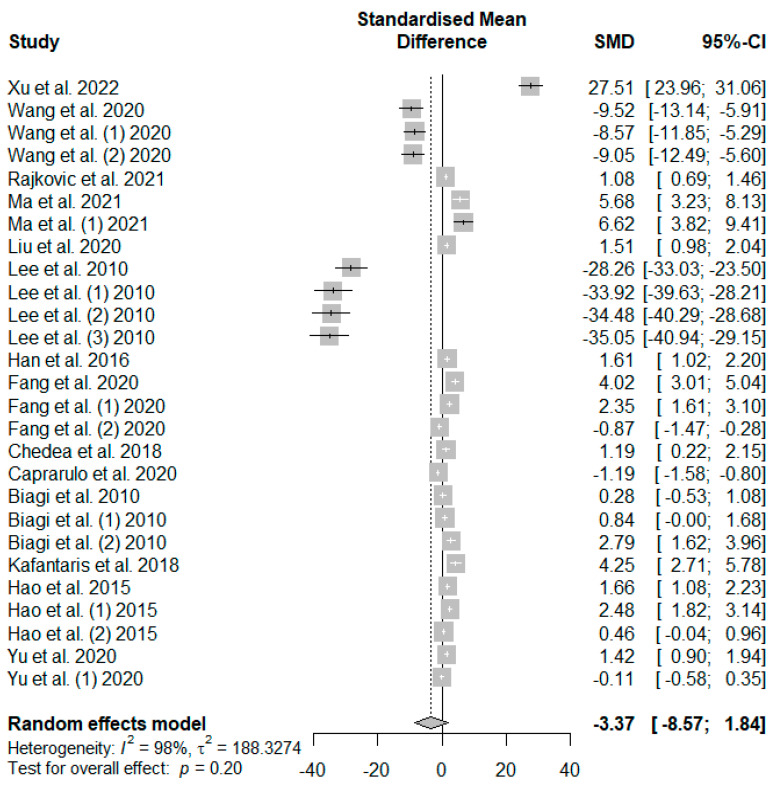
Forest plot of the effect size or standardized mean difference and 95% confidence interval of tannins on weaned piglets’ average daily gain (ADG). The solid vertical black line represents the mean difference of zero or no effect. Points to the left of the solid vertical black line represent a reduction in ADG, while points to the right of the solid line indicate an increase in ADG.

The effects of tannin supplementation on the growth performance of weaned piglets were summarized using random-effects models of meta-analysis. Figure 3 summarizes the meta-analysis on the impact of tannin supplementation on the average daily gain (ADG) of weaned piglets. A non-significant decreasing effect (*P*_SMD_ = 0.20) of tannin supplementation in weaned piglets was observed for ADG.

**Figure 4:**
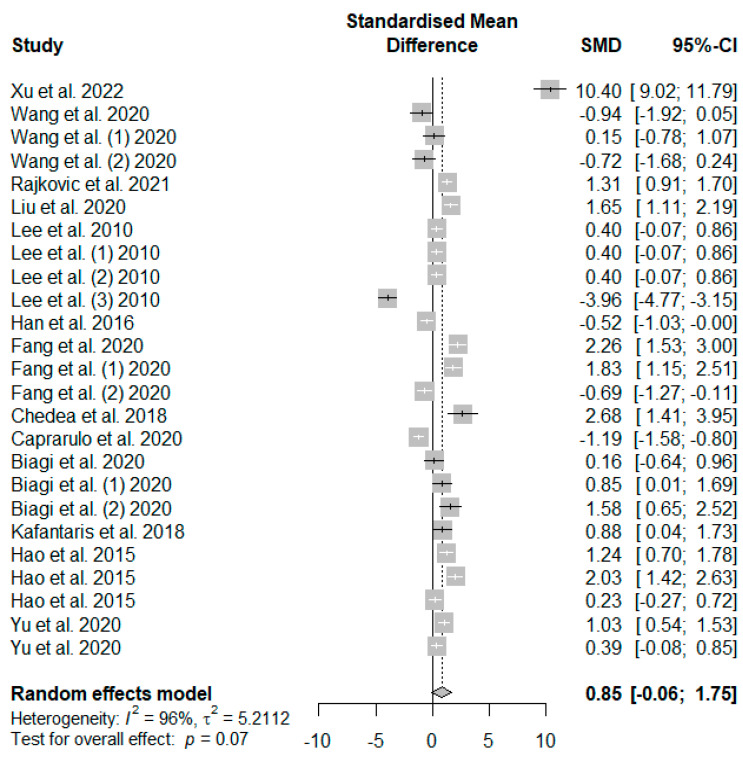
Forest plot of the effect size or standardized mean difference and 95% confidence interval of tannins on weaned piglets’ average daily feed intake (ADFI). The solid vertical black line represents the mean difference of zero or no effect. Points to the left of the solid vertical black line represent a reduction in ADFI, while points to the right of the solid line indicate an increase in ADFI.

The summary of the meta-analysis on the effects of tannin supplementation on the average daily feed intake (ADFI) of weaned piglets is shown in Figure 4. Supplementation with tannins did not significantly (*P*_SMD_ = 0.07) affect weaned piglets’ ADFI.

**Figure 5:**
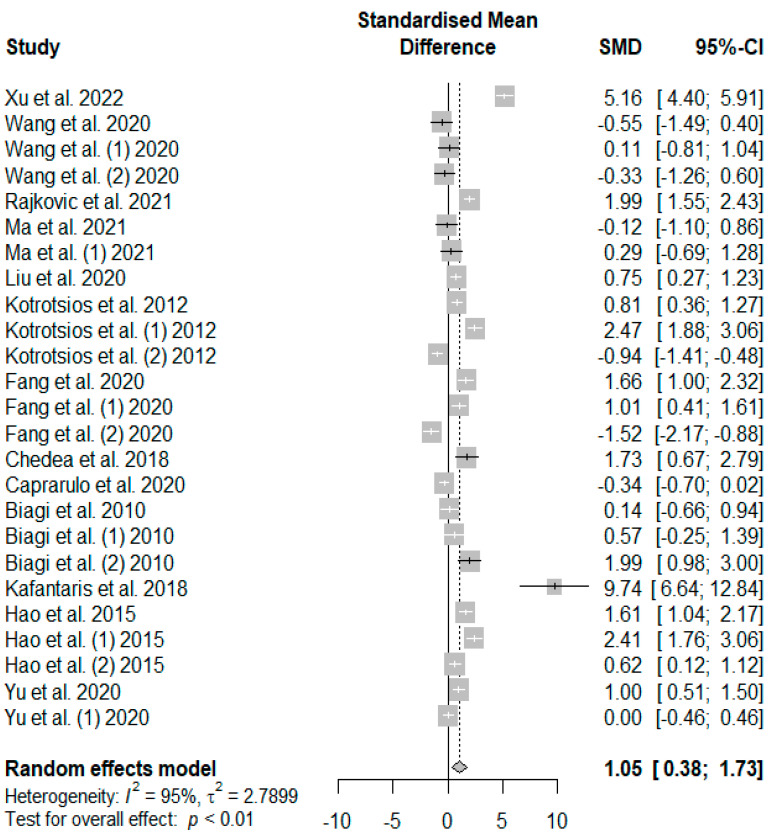
Forest plot of the effect size or standardized mean difference and 95% confidence interval of tannins on weaned piglets’ final body weight (FBW). The solid vertical black line represents the mean difference of zero or no effect. Points to the left of the solid vertical black line represent a reduction in FBW, while points to the right of the solid line indicate an increase in FBW.

Figure 5 summarizes the meta-analysis on the effects of tannin supplementation on the final body weight (FBW) of weaned piglets. Tannin supplementation significantly increased FBW (SMD = 1.05, *P*_SMD_ < 0.01, *I*^2^ = 95%), with the observed overall effect size (SMD = 1.05) over and above the SMD value of 0.7, suggesting a high tannin effect size on FBW.

**Figure 6:**
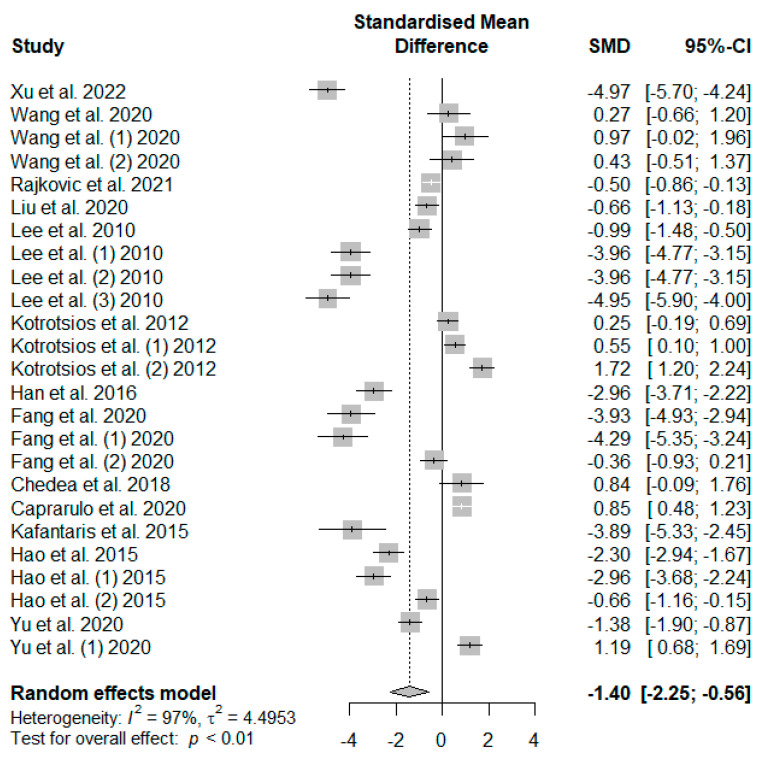
Forest plot of the effect size or standardized mean difference and 95% confidence interval of tannins on weaned piglets’ feed conversion ratio (FCR). The solid vertical black line represents the mean difference of zero or no effect. Points to the left of the solid vertical black line represent a reduction in FCR, while points to the right of the solid line indicate an increase in FCR.

Figure 6 presents the effect of tannin supplementation on the feed conversion ratio (FCR) of weaned piglets. Supplementing the basal diets of weaned piglets with dietary tannins significantly decreased FCR (SMD = −1.40, *P*_SMD_ < 0.01, *I*^2^ = 97%). The observed overall effect size was shown to be above the SMD value of 0.70, reflecting a high tannin effect size on piglets’ FCR.

#### 3.3.2. Serum Antioxidant Parameters

**Figure 7:**
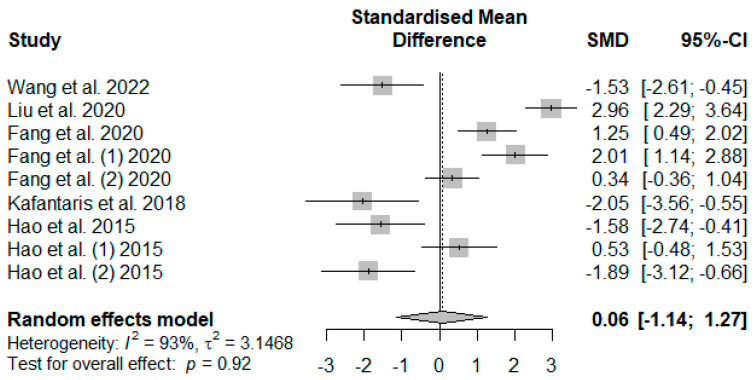
Forest plot of the effect size or standardized mean difference and 95% confidence interval of tannins on weaned piglets’ serum catalase (CAT). The solid vertical black line represents the mean difference of zero or no effect. Points to the left of the solid vertical black line represent a reduction in CAT, while points to the right of the solid line indicate an increase in CAT.

The dietary tannin supplementation effects on weaned piglets’ serum antioxidant indices were summarized using random-effects models of meta-analysis. Figure 7 illustrates the summary of the meta-analysis on the impact of tannin supplementation on the serum catalase (CAT) of weaned piglets. There was no significant (*P*_SMD_ = 0.92) effect of tannin supplementation on serum CAT.

**Figure 8:**
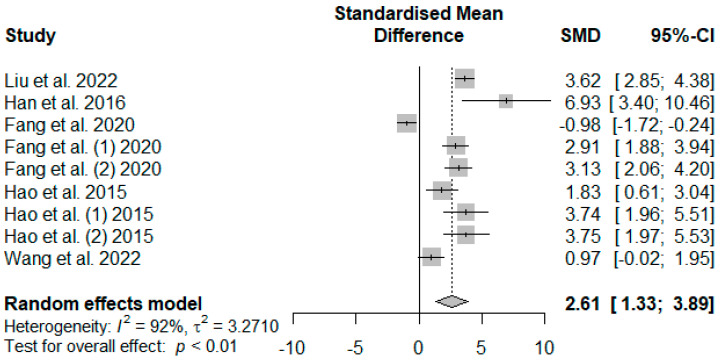
Forest plot of the effect size or standardized mean difference and 95% confidence interval of tannins on weaned piglets’ serum superoxide dismutase (SOD). The solid vertical black line represents the mean difference of zero or no effect. Points to the left of the solid vertical black line represent a reduction in SOD, while points to the right of the solid line indicate an increase in SOD.

Figure 8 presents the effect of tannin supplementation on weaned piglets’ serum superoxide dismutase (SOD). Supplementing the basal diets of weaned piglets with tannins significantly increased SOD (SMD = 2.61, *P*_SMD_ < 0.01, *I*^2^ = 92%), suggesting a high (SMD > 0.70) tannin treatment effect.

**Figure 9:**
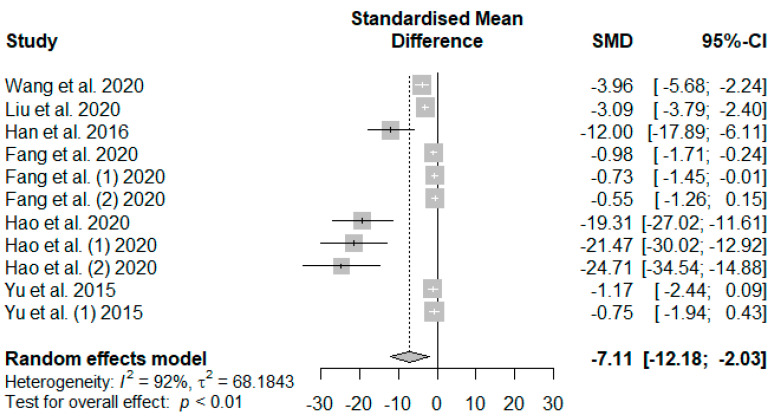
Forest plot of the effect size or standardized mean difference and 95% confidence interval of tannins on weaned piglets’ serum malondialdehydes (MDAs). The solid vertical black line represents the mean difference of zero or no effect. Points to the left of the solid vertical black line represent a reduction in MDA, while points to the right of the solid line indicate an increase in MDA.

The summary of the meta-analysis on the effects of tannin supplementation on weaned piglets’ serum malondialdehydes (MDAs) is shown in Figure 9. The overall effect of the analysis indicated a significant decreasing impact (SMD = −7.11, *P*_SMD_ < 0.01, *I*^2^ = 92%) of tannins when supplemented in the basal diet of weaned piglets. The effect size suggests a high (SMD > 0.70) tannin effect on serum MDAs.

**Figure 10:**
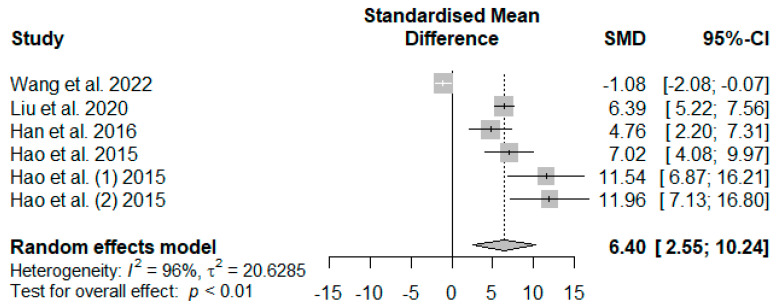
Forest plot of the effect size or standardized mean difference and 95% confidence interval of tannins on weaned piglets’ serum glutathione peroxidase (GSH-Px). The solid vertical black line represents the mean difference of zero or no effect. Points to the left of the solid vertical black line represent a reduction in GSH-Px, while points to the right of the solid line indicate an increase in GSH-Px.

Figure 10 illustrates the summarized meta-analysis effects of supplemented tannins on weaned piglets’ serum glutathione peroxidase (GSH-Px). The overall impact of the analysis indicated a significant increase in the effect (SMD = 6.40, *P*_SMD_ = 0.01, *I*^2^ = 96%) of tannin supplementation. The effect size (SMD > 6.40) reveals that tannins highly affected serum GSH-Px.

**Figure 11:**
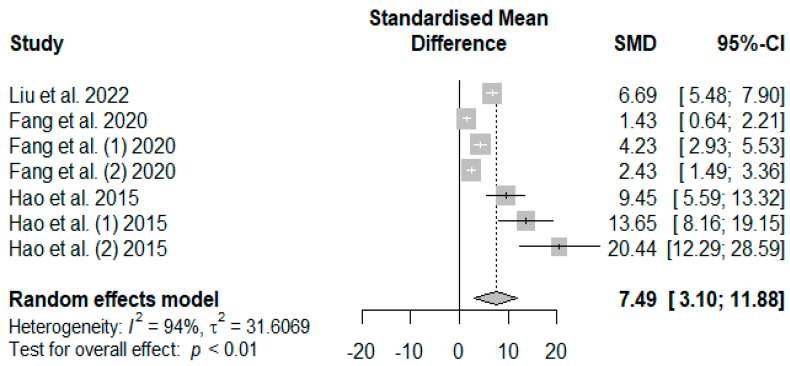
Forest plot of the effect size or standardized mean difference and 95% confidence interval of tannins on weaned piglets’ serum total antioxidant capacity (T-AOC). The solid vertical black line represents the mean difference of zero or no effect. Points to the left of the solid vertical black line represent a reduction in T-AOC, while points to the right of the solid line indicate an increase in T-AOC.

The summary of the meta-analysis on the effects of tannin supplementation on weaned piglets’ serum total antioxidant capacity (T-AOC) is shown in Figure 11. The overall impact of the analysis revealed a significantly increased effect (SMD = 7.49, *P*_SMD_ < 0.01, *I*^2^ = 94%) of tannins when supplemented in the basal diet of weaned piglets. The effect size (SMD > 0.70) can be considered a high tannin effect on serum T-AOC.

#### 3.3.3. Immune Indices

**Figure 12:**
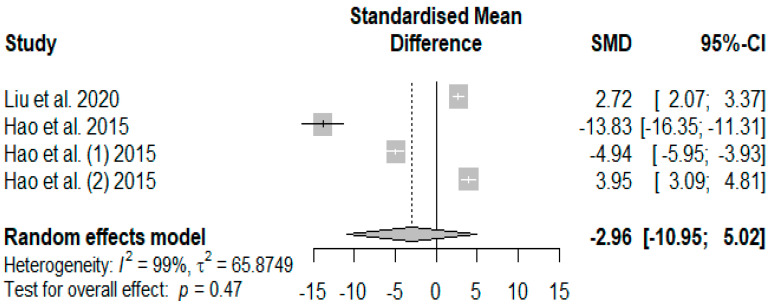
Forest plot of the effect size or standardized mean difference and 95% confidence interval of tannins on weaned piglets’ serum immunoglobin A (IgA). The solid vertical black line represents the mean difference of zero or no effect. Points to the left of the solid vertical black line represent a reduction in IgA, while points to the right of the solid line indicate an increase in IgA.

The influence of supplementing tannins on weaned piglets’ serum immune indices was summarized using random-effects models of meta-analysis. Figure 12 presents the overall effects of supplemented tannins on weaned piglets’ immunoglobin A (IgA). A non-significant decreasing effect (*P*_SMD_ = −2.96) was observed for IgA.

**Figure 13:**
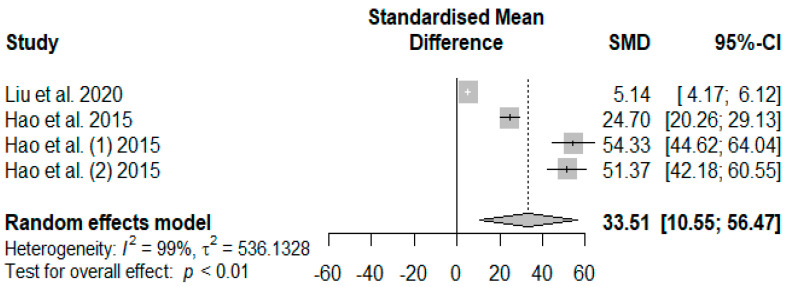
Forest plot of the effect size or standardized mean difference and 95% confidence interval of tannins on weaned piglets’ serum immunoglobin M (IgM). The solid vertical black line represents the mean difference of zero or no effect. Points to the left of the solid vertical black line represent a reduction in IgM, while points to the right of the solid line indicate an increase in IgM.

The summary of the meta-analysis on the effects of tannin supplementation on weaned piglets’ serum immunoglobin M (IgM) is shown in Figure 13. The overall impact of the analysis revealed a significantly increased effect (SMD = 33.51, *P*_SMD_ < 0.01, *I*^2^ = 99%) of tannins when supplemented in the basal diet of weaned piglets. The effect size (SMD > 0.70) can be considered a high tannin effect on serum IgM.

**Figure 14:**
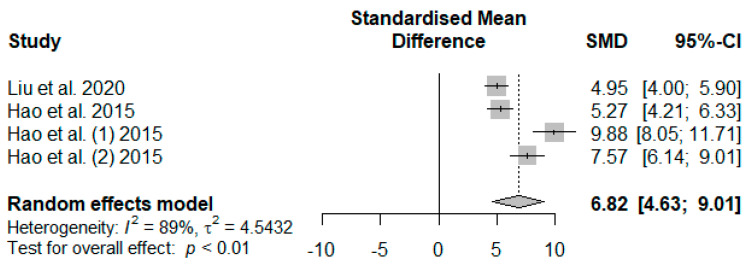
Forest plot of the effect size or standardized mean difference and 95% confidence interval of tannins on weaned piglets’ serum immunoglobin M (IgG). The solid vertical black line represents the mean difference of zero or no effect. Points to the left of the solid vertical black line represent a reduction in IgG, while points to the right of the solid line indicate an increase in IgG.

Figure 14 summarizes the meta-analysis on the effects of tannin supplementation on the immunoglobin G (IgG) of weaned piglets. Tannin supplementation significantly increased IgG (SMD = 6.82, *P*_SMD_ < 0.01, *I*^2^ = 89%), indicating a high tannin treatment effect (SMD > 0.70).

### 3.4. Subgroup Analysis of Tannin Source Effects on Weaned Piglets’ Growth Performance

Subgroup analysis, a form of moderator analysis and considered a special case meta-regression by Thompson and Higgins [66], examines the impact of a single categorical variable. Subgroup analysis was performed to test the hypothesis that some tannin sources yield higher effects than others (i.e., studies in our meta-analysis do not stem from one overall population), assuming that they fall into different subgroups and that each subgroup has its actual overall effect. Although all parameters assessed in our meta-analysis yielded *I*^2^ ≥ 75%, subgroup analyses were only performed for parameters with (K) ≥ 10 studies. Borenstein et al. [62] (2015) mentioned, as a general rule of thumb, that subgroup analysis only makes sense when the meta-analysis contains at least ten studies. Table 2 presents the outcome of the tannin source subgroup analysis on average daily feed intake (ADFI), average daily gain (ADG), final body weight (FBW), and feed conversion ratio (FCR). Using the value of X^2^ statistics (*Q*) to determine whether the subgroup differences were significant enough to not be explainable by sampling error alone, the observed value of *Q* was substantially more significant than the expected one, indicating that there was a significant difference (*p* < 0.0001) in the actual effect sizes between subgroups.

Regarding weaned piglets’ ADFI, supplementation with chestnut, grape extract, grape seed proanthocyanidins (GSP), grape pomace, and gallnut tannic acid had a positive effect, with chestnut recording the highest increasing effect (SMD = 2.9020), followed by grape extract (SMD = 1.3074) and grape seed proanthocyanidins (SMD = 0.8966). Conversely, the gallnut microencapsulated tannic acids (SMD = −0.5921) and chestnut and quebracho blend (SMD = −1.1924) adversely affected ADFI. Similarly, weaned piglets’ ADG increased when tannins were sourced from chestnut (SMD = 6.4894), grape extract (SMD = 1.0764), grape seed proanthocyanidins (SMD = 1.6444), grape pomace (SMD = 2.6567), quebracho (SMD = 6.0871), and gallnut tannic acid (SMD = 0.6481). Chestnut again registered the optimum increasing effect on ADG. In contrast, ADG was decreased by gallnut microencapsulated tannic acid and the chestnut and quebracho blend (Table 2), with GMTA having a considerable influence. The final body weight increased in weaned piglets supplemented with grape pomace (SMD = 5.5956), grape extract (SMD = 1.9873), chestnut (SMD = 1.7184), GSP (SMD = 0.9624), carob pods (SMD = 0.7726), gallnut tannic acid (SMD = 0.4981), and quebracho (SMD = 0.0868), but decreased when supplemented with the chestnut and quebracho blend (SMD = −0.3416) and GMTA (SMD = −0.2500). Conversely, the effect trends of the respective tannin sources on ADFI, ADG, and FBW were opposite in the piglets’ feed conversion ratio (FCR). The piglets’ FCR decreased with supplementation with chestnut (SMD = −2.8044), GSP (SMD = −2.4528), GMTA (SMD = −1.7433), grape pomace (SMD = −1.4908), grape extract (−0.4968), and gallnut tannic acid (SMD = −0.0982) but increased with a blend of chestnut and quebracho (SMD = 0.8517) and carob pods (SMD = 0.8295). Considering the high heterogeneities (*I*^2^ ≥ 75%) detected among the growth outcomes in our global studies (meta-analysis), it was revealed in the subgroup analysis that the chestnut subgroup contributed the most to the heterogeneity.

### 3.5. Effect of Tannin Dosage, Supplementation Duration, and Piglets’ Weaned Age on Growth Performance

**Figure 15:**
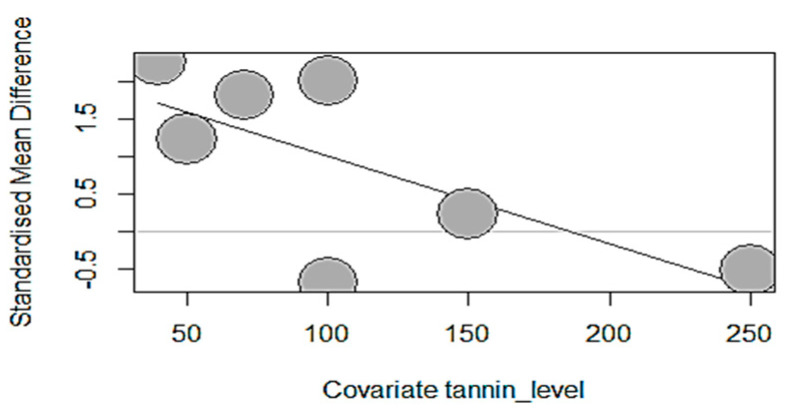
Effect of GSP dosage on ADFI showing a decreasing trend of ADFI’s standardized mean difference following the increasing dosage of GSP supplementation.

Like the subgroup analysis, meta-regression analysis, a form of moderator analysis that accommodates both continuous and categorical moderators, was only performed for growth-measured outcomes (number of studies, k ≥ 10). Borenstein et al. [67] mentioned that this guideline may also be applied to meta-regression models but should not be considered an iron-clad rule. Therefore, the present meta-analysis only applied meta-regression to GMTA, GSP, chestnut, and carob pods. To investigate what caused the patterns of heterogeneity in our data, three meta-regressions with tannin source dosage, supplementation duration, and piglets’ age as predictors were performed. The tannin source dosage effects on piglets’ growth performance parameters are displayed in Table 3. The results of the meta-regression models indicated a non-significant (*p* > 0.05) tannin source effect for ADG, FBW, and FCR except for grape seed proanthocyanidins, which significantly decreased ADFI (estimate = −0.0118, TM = 4.7277, *p* = 0.0297). The dosage of GSP supplementation explained 40.74% of the observed heterogeneity for ADFI. This observation is highlighted by the bubble plot (Figure 15), which showed a significant negative impact of GSP due to its increasing dosage (40–250 mg/kg).

**Figure 16:**
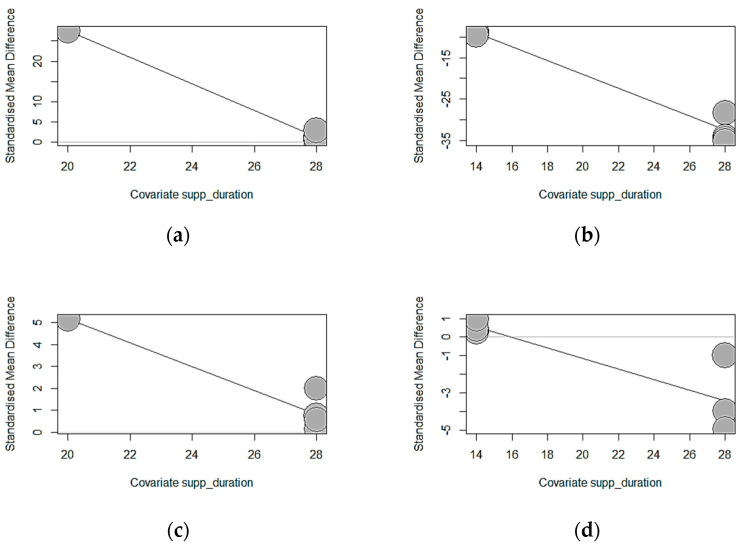
Showing a decreasing trend of standardized mean difference following the increasing dosage of tannin supplementation duration on weaned piglets’ growth performance: (**a**) impact of chestnut on ADG; (**b**) impact of GMTA on ADG; (**c**) impact of chestnut on FBW; (**d**) impact of GMTA on FCR.

Table 4 presents the meta-regression of the tannin source supplementation duration effects on the growth performance of weaned piglets. Except for ADFI, significant correlation results were found for ADG (*p* < 0.0001), FBW (*p* < 0.0001), and FCR (*p* = 0.0002). Longer exposure of chestnut (estimate = −3.2762, TM = 158.2981) and GMTA (estimate = −1.6715, TM = 183.7752) significantly decreased ADG. The duration of GMTA supplementation explained the observed heterogeneity thoroughly (100%), whereas the chestnut supplementation duration explained 99.40%. Similarly, increasing the supplementation duration of chestnuts reduced the final body weight (estimate = −1.6715, TM = 183.7752, *p* < 0.0001) of weaned piglets. Longer chestnut supplementation explained the observed heterogeneity thoroughly (100%). Equally, GMTA supplementation for a longer duration significantly lowered the FCR (estimate = −0.2836, TM = 13.6825, *p* = 0.0002) of weaned piglets, explaining 69.89% of the observed heterogeneity for FCR. The significant results recorded for ADG, FBW, and FCR according to the duration of supplementation are highlighted by the bubble plots in Figure 16.

Table 5 shows the meta-regressions of piglets’ age effects on the tannic source and growth-measured outcome association. The covariate piglet age had no significant relationship (*p* > 0.05) with ADFI, ADG, FBW, or FCR.

**Figure 17:**
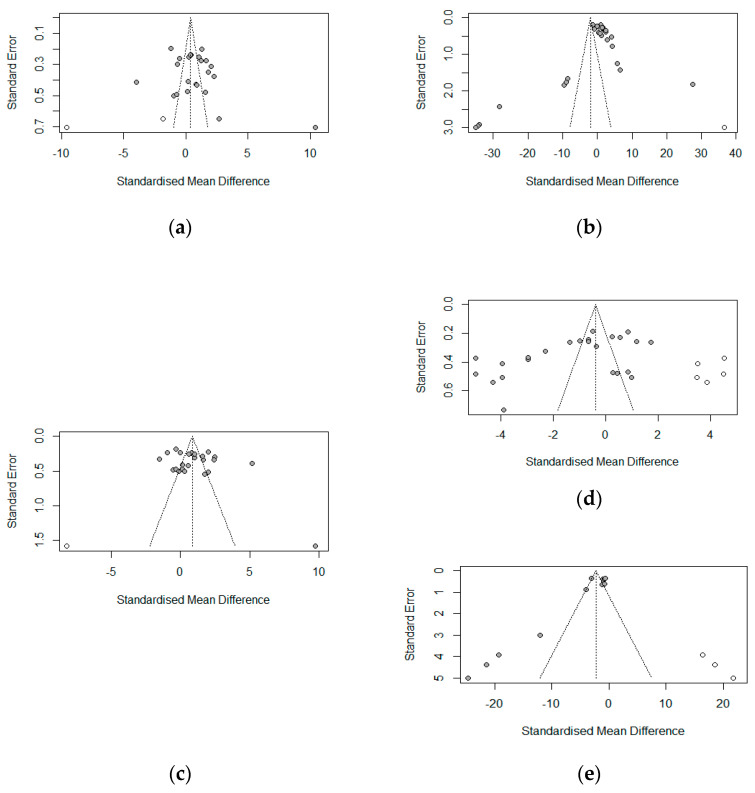
Funnel plot assessment for publication bias on weaned piglets’ growth outcomes and serum malondialdehydes: (**a**) ADFI; (**b**) ADG; (**c**) FBW; (**d**) FCR; (**e**) MDA.

Figure 17 shows the funnel plot assessment of publication bias for the growth parameters (ADFI, ADG, FBW, FCR) and malondialdehydes (serum antioxidant index) via the grey data points. The funnel plots indicated no evidence of asymmetry (publication bias) for ADFI (*p* = 0.1908), ADG (*p* = 0.2340), or FBW (*p* = 0.1912), which was confirmed by Egger’s linear test for publication bias (Table 6). FCR and MDA, on the other hand, showed evidence of asymmetry. These results were confirmed by Egger’s linear regression test (Table 6) for FCR (bias = −9.8262, *p* = 0.0031) and MDA (bias = −4.3594, *p* = 0.0048).

## 4. Discussion

A wide range of physiological responses, such as impaired intestinal metabolism and function, compromised immunity, and decreased antioxidant capacity, which lead to depressed feed utilization, retarded growth, and increased morbidity and mortality, are associated with the phase of weaning [33]. Tannins, traditionally regarded as an “anti-nutritional” factor, negatively impact feed intake, nutrient digestibility, and production performance in monogastric nutrition [68,69]. Nonetheless, reports of several recent studies indicate that low concentrations of tannins from several sources improved nutrition, animal performance, and health status in monogastric animals [22,25,70,71]. Our present meta-analysis, therefore, investigated the tannin source effects on weaned piglets’ growth performance, serum antioxidant capacity, and immune status.

### 4.1. Study Quality Assessment

The quality of evidence in a systematic review (SR) is as important as analyzing the data. The quality and reliability of a systematic review’s evidence, according to Macleod et al. [72], is linked closely to the credibility of the data and the results of the individual studies included. Therefore, assessing the risk of bias, a measure of each study’s quality, is vital for SR. The included studies in our review were published between 2010 and 2023; this characteristic aligns with the surge in the call for the adoption and use of similar reporting standards in the description of the methods section of real-world animal intervention data studies. However, in our present meta-analysis, 37% of the included studies showed an unclear risk of bias. This observation of bias is instituted by inadequate randomization and a lack of blinding, allocation concealment, and sequence, which are not yet standard practices in animal intervention experimentation. This assertion is supported by Macleod et al. [65] and Kilkenny et al. [73], who indicated that most individual studies do not report these measures and are not a common practice in animal experiments. Despite the aggregated percentage of unclear risk of bias, the included studies were of low risk of bias (53%) even with a 10% high risk of bias. The findings of the risk assessment of individual studies’ methodological, reporting, and evidence quality of the MA of tannin supplementation in the basal diet of weaned piglets revealed that the quality of evidence was valid, though not devoid of bias.

### 4.2. Growth Performance

Tannins, an astringent group of polyphenolic compounds, develop complexes with other nutrients, such as proteins, minerals, or digestive enzymes, which are capable of reducing feed palatability, feed intake, and nitrogen digestibility in monogastric species, including growing pigs [28,74,75]. Conversely, in the present meta-analysis, increased ADFI and FBW and a reduced FCR in weaned piglets were observed in response to dietary supplementation with different tannins. This observation implies that tannins are generally a potential nutritional source for improving growth and feed utilization in weaned piglets.

This observation is supported by the assertion that pigs, compared to other domestic animals, appear to be relatively resistant to the presence of tannins in their diets, as they are capable of consuming relatively high amounts of tannin-rich feeds without presenting symptoms of toxicity [76]. This is likely due to parotid gland hypertrophy and the secretion of proline-rich proteins in their saliva. These types of proteins can bind and neutralize the toxic effects of tannins [77,78]. Medium supplementation of tannins seems to be more effective on the growth of post-weaned piglets than low inclusion [22,25,79,80,81]. Yet, some studies indicated an astringent sensation produced by a reaction between dietary tannins and salivary mucoproteins or by a direct response from tannins with taste receptors, manifesting a reduction in palatability and thereby reducing feed intake [82,83]. The observed increased ADFI and FBW in response to tannins, despite the reduced ADG (possibly due to poor protein digestibility of the tannin-rich diet), could be explained by tannin resistance associated with elevated synthesis of proline-rich proteins (PRPs) in the saliva, which bind tannins from feedstuffs and prevent intoxication of organisms with diets rich in tannins [84,85]. In addition, prolonged exposure to dietary tannins can induce adaptive mechanisms in the amount of proline and other salivary proteins with a high affinity for tannins [83]. Dietary tannin complexes formed with macromolecules and protein complexes rich in proline, unlike other complexes, are stable over the entire pH range of the digestive tract, which could increase the efficiency of nutrient utilization of the diet [86] and also eliminate or reduce their negative effect on palatability and feed intake [82,87].

Regarding these explanations, in our meta-analysis, tannin sources except GMTA and the blend of chestnut and quebracho (Ch/Qu) supplemented according to our sub-group analysis (Table 2) positively influenced response in ADFI and FBW. The reducing effects of GMTA and Ch/Qu blend could be attributed to their poor palatability, which possibly accounts for ADFI and feed efficiency depression when supplemented in weaned piglets’ diet [23,27,36,88]. Although the exact mechanisms of how tannins improve performance are not fully understood in monogastric species [40], the heterogeneity observed for ADFI (Table 3 and Table 4) in our meta-analysis was partly explained by tannin source dosage (GSP = 40.74%, GMTA = 6.01%), whereas 12.37% by GMTA supplementation for ADG. On the other hand, the duration of chestnut and GMTA supplementation explained 99.40% and 100% of the heterogeneity in ADG, respectively. Similarly, the observed heterogeneity in FBW can be explained by chestnut supplementation duration. Even though it has been previously reported that the impact of tannins depends on the dose of the tannins in the diet rather than the type of tannins [89], the results of our present sub-group analysis and meta-regression suggest that the effectiveness of tannin supplementation in weaned piglets seems to be highly related not only to the dosage of tannins administered but also the tannin source and its supplementation duration. This observation affirms the finding of Bueno et al. [90], who reported that the same dose of tannins from different sources can influence nutrient availability differently. In addition, according to Mueller-Harvey [75], tannin source biodegradation and absorption along the gastrointestinal tract are influenced by the type of tannin it possesses, with the fate of condensed tannins seeming to be more complex due to their structural complexity.

Concerning FCR, the general reducing effects of dietary tannin sources in our meta-analysis could be due to the activity of tannins’ bioactive compounds, which modulate intestinal metabolism, protect intestinal morphology health, and reduce the incidence of intestinal disease, thus enhancing piglets’ performance. This claim is reinforced by that of Huang, Liu, Zhao, Hu, and Wang [23], who said that supplementing tannins tends to inhibit the progression of infections in the gastrointestinal tract, thereby improving feed efficiency in livestock. Likewise, tannins can positively influence gut morphology and enhance nutrient absorption in monogastric animals [91]. The beneficial effects of phytobiotics, comprising tannins, include not only anti-bacterial activities, antioxidation, and immuno-modulation but also nutrient digestion, absorption, and stimulation of intestinal mucus secretion, saliva, and bile [92,93]. The influence of the antioxidant property of tannins on enzyme activity may partly explain the reduced phenomenon of FCR. Antioxidant enzymes and exogenous antioxidants can help restore oxidative balance and maintain healthy intestinal mucosa, increasing nutrient absorption [62,94,95]. The FCR observed suggests a positive effect of tannin supplementation on the economics of gain in swine production. Consequently, the effectiveness of dietary sources of tannins on the productive performance of weaned piglets is more pronounced and positive using chestnut and grape seed proanthocyanidins.

Even though some publication bias was found on FCR, statistically, the decreasing effect of tannins observed in our first result (Table 6) did not change when the corrected publication bias test (Table 7) was performed. Therefore, the reduction effect of tannins on FCR will be considered in the present meta-analysis as the actual effect. This assertion is validated in our sub-group analysis (Table 2), where all tannin sources except carob pods and Ch/Qu decreased FCR response.

### 4.3. Serum Antioxidant Capacity

Animal nutritionists have directly explored the antioxidant properties of polyphenolic compounds as preservatives in compound feed to protect animals against the harmful consequences of feed component oxidation [96]. According to Ghiselli et al. [97], exogenous antioxidants establish the first line of defense against excessive reactive oxygen species (ROS) generation to protect the organism against harmful peroxidation. In our meta-analysis, excessive accumulation of this prooxidant substance is hypothesized to cause oxidative stress in weaned piglets. Inhibiting this oxidative stress in animals and stabilizing their products’ antioxidant potential (e.g., meat and eggs) can be achieved with polyphenolic compounds [96]. Although these effects are triggered by simple phenolics, tannins (CT and HT) exhibited more excellent antioxidant activity due to their relatively high molecular weight [98].

In the present study, serum CAT, SOD, GSH-Px, and T-AOC were observed to generally increase in response to dietary supplementation with tannins of different sources. These observations hint at enhanced antioxidant enzyme activities in the serum of weaned piglets, thereby reducing their susceptibility to lipid peroxidation. The findings of this study are confirmed by those of [32,33,62,96], who reported tannin reduction effects on CAT, SOD, GSH-Px, and T-AOC in the serum of weaned piglets. These observations could be partly accounted for by tannins’ moderate bioavailability and influence on absorption. Supplementation with antioxidant-rich foods or purified antioxidants yielding subsequent changes in blood plasma provides information on the absorption and bioavailability of ingested antioxidant compounds [97]. Although the exact metabolic mechanisms of polyphenols, including tannins, have not been fully elucidated in pigs [99], the results observed in our meta-analysis imply that tannins ingested by weaned piglets may be degraded and absorbed in the gastrointestinal tract and subsequently transferred to the bloodstream to serve as exogenous antioxidants. Another possible reason for these findings is that tannins can selectively induce antioxidant enzyme gene expression via modulating redox-sensitive signaling pathways by inhibiting lipid peroxidation and quenching the oxygen free radicals in the gut [100]. The elevated levels of CAT, SOD, and GSH-Px are vital in converting ROS into less harmful compounds and consequently reducing ROS-mediated damage on DNA and entire chromosomes, amino acid modification, contribution to protein fragmentation, lipid peroxidation intensification in cell membranes, and cell apoptosis and necrosis which increase the risk of inflammations and cancer [99,101,102]. The trim-and-fill model results (Table 7) confirmed the global results (Figure 7, Figure 8, Figure 10, and Figure 11) of the present meta-analysis by showing positive effect sizes for CAT, SOD, GSH-Px, and T-AOC, suggesting that supplementation with tannins minimizes the negative consequences of oxidative stress during weaning.

In the present meta-analysis, MDA in weaned piglets’ serum was generally reduced in response to dietary tannin supplementation. The finding of this meta-analysis showed that tannins mitigate oxidative stress, which are similar to the findings of [103,104], who reported a decrease in blood MDA level when broilers and piglets were supplemented with polyphenolic compounds. MDA can induce toxic stress in cells and constitute homopolar protein adducts identified as advanced lipoxidation end-products (ALEs), a marker for oxidant stress-level estimation [105]. Their reduction in this study suggests that tannins supplemented in the diet of weaned piglets could be utilized as a dietary strategy to alleviate oxidative stress. Even though the reducing effects of tannins on MDA in the global study (Figure 9) of our meta-analysis were influenced by publication bias (Table 6), the decrease in MDA levels was an actual influence (Table 7) of the source of the dietary tannins in our meta-analysis.

### 4.4. Immune Indices

So far, the immunomodulatory effects of polyphenolic compounds in swine, including tannins, are explained mainly by their anti-oxidant activities [23,42]. Tannins are highlighted for their immunity-enhancing impact on the gut by inhibiting bacterial growth or bacterial adhesion to the intestinal epithelium and biofilm formation and reducing damage to the intestine via enterotoxin production and activity inhibition [42]. Under oxidative stress, particularly in piglets, polyphenols can enhance immunity by activating immunoglobulins and inhibiting the secretion of proinflammatory cytokines [99].

In the present meta-analysis, the immune effect of tannin supplementation is emphasized by the increase in IgG and IgM serum levels, signifying their suppressive effects on intestinal inflammations and possibly reducing the incidence of diarrhea. These findings are affirmed by Cappelli et al. [106] and Verhelst et al. [101], who explored polyphenols from other sources and concluded their effectiveness in decreasing the incidence of diarrhea in piglets. A possible reason that could explain this phenomenon is the ability of polyphenols, including tannins, to lower the activity of inflammatory mediators NF-κB and Nrf2 through inhibition of the NF-κB/P38 signaling pathways [106,107], thus reducing the risk of intestinal diseases [108].

The global immunomodulation effects (Figure 12, Figure 13 and Figure 14) of dietary supplementation with tannins in our present meta-analysis suggest that tannins are a potential nutritive additive to mitigate inflammatory mediators. The observed global immunomodulatory effects in our meta-analysis were further confirmed in the trim-and-fill test (Table 7).

## 5. Conclusions

The present meta-analysis evaluated the effect of dietary supplementation with tannins on weaned piglets’ growth performance, serum antioxidant capacity, and serum immune status. Supplementing tannins improved average daily feed intake, final body weight, and the feed conversion ratio. The enhancement of weaned piglets’ productive performance due to tannins depended on the source, dosage, and duration of exposure. Likewise, dietary supplementation with tannins effectively reduced malondialdehydes. Still, they elevated the levels of glutathione peroxidase, superoxide dismutase, and total antioxidant capacity in the serum, which, in general, could be a reliable nutritional strategy for oxidative stress alleviation. The evidence from the meta-analysis also indicates a tannin-related increase in immunoglobin G and M. Supplementing weaned piglets’ diet with tannins, particularly with chestnut and grape seed proanthocyanidins, could be a reliable nutritional strategy for improving and sustaining gut health, which could translate into mitigating diarrhea occurrences. With the pronounced effect of chestnut and grape seed proanthocyanidins on weaned piglets’ performance, further investigation of their response to the exposure interaction of dosage duration would be fascinating.

## Figures and Tables

**Table 1:** Summary of characteristics of studies included in the meta-analysis.

Author (Year)	Weaned Age (Days)	Suppl. Duration (Days)	Tannin Type ^1^	Tannin Source ^2^	Average Weight (kg)	Tannin Dosage (mg/kg)	Parameters of Analysis ^3^
Xu et al. [60]	28	20	HT	Chestnut	8.6	1500	FBW, ADFI, ADG, FCR
Wang et al. [38]	21	14	HT	GMTA	5.99 ± 0.13	500, 1000, 1500	FBW, ADFI, ADG, FCR
Wang et al. [61]	21	14	HT	GMTA	5.99 ± 0.13	1000	GSH-Px, SOD, CAT, MDA
Rajkovic et al. [15]	23	56	CT	Grape extract	6.9 ± 0.1	148	FBW, ADFI, ADG, FCR
Ma et al. [37]	28	20	CT	Quebracho	-	2000, 3000	FBW, ADG
Liu et al. [62]	28	28	HT	Chestnut wood	7.81 ± 0.99	1000	FBW, ADFI, ADG, FCR, T-AOC, SOD, GSH-Px, CAT, MDA, IgA, IgG, IgM
Lee et al. [36]	21	28	HT	G. tannic acid	6.485 ± 0.66	125, 250, 500, 1000	ADFI, ADG, FCR
Kotrotsios et al. [35]	30	55	CT	Carob pods	-	75,000, 100,000,125,000	FCR
Han et al. [63]	28	28	CT	GSP	9.3 ± 0.5	250	ADFI, ADG, FCR, SOD, GSH-Px, MDA
Fang et al. [32]	28	28	CT	GSP	8.4 ± 0.17	40, 70, 100	FBW, ADFI, ADG, FCR, T-AOC, SOD, GSH-Px, CAT, MDA
Chedea et al. [64]	21	36	CT	Grape pomace	10.70 ± 0.8	50,000	FBW, ADFI, ADG, FCR
Caprarulo et al. [31]	35	40	HT/CT	blend (Ch/Qu)	-	12,500	FBW, ADFI, ADG, FCR
Biagi et al. [22]	28	28	HT	Chestnut wood	8.23 ± 0.93	1130, 2250, 4500	FBW, ADFI, ADG
Kafantaris et al. [34]	21	30	CT	Grape pomace	6.49 ± 0.66	90,000	FBW, ADFI, ADG, FCR, GSH-Px, CAT
Hao et al. [33]	21	28	CT	GSP	6.99 ± 0.11	50, 100, 150	FBW, ADFI, ADG, FCR, T-AOC, SOD, GSH-Px, CAT, MDA, IgA, IgG, IgM
Yu et al. [65]	21	28	HT	G. tannic acid	6.6 ± 0.27	1973.09, 12,004.84	FBW, ADFI, ADG, FCR, MDA

^1^ CT: condensed tannin; HT: hydrolyzable tannin. ^2^ GMTA: gallnut microencapsulated tannic acid; GSP: grape seed proanthocyanidins; Ch/Qu: chestnut/quebracho; G. tannic acid: gallnut tannic acid. ^3^ ADG: average daily gain; ADFI: average daily feed intake; FBW: final body weight; FCR: feed conversion ratio; MDA: malondialdehydes; SOD: superoxide dismutase; CAT: catalase; GSH-Px: glutathione peroxidase; T-AOC: total antioxidant capacity; IgA: immunoglobulin A; IgG: immunoglobin G; IgM: immunoglobin M.

**Table 2:** Meta-ANOVA of the association between tannin source and measured growth outcomes.

Variable	Random-Effects Model				Heterogeneity					
			95% CI							*p*-Value
	k	SMD	Lower	Upper	*I* ^2^	τ^2^	τ	Q	df	
***ADFI*, g/day**										
Chestnut (Ch)	5	2.9020	−0.7572	6.5612	97.6	17.1981	4.1471	90.69	6	<0.0001
GMTA	7	−0.5921	−1.7603	0.5761	94.3	2.3387	1.5293			
Grape extract	1	1.3074	0.9121	1.7027	--	--	--			
GSP	7	0.8966	−0.0135	1.8067	93.8	1.4162	1.1901			
Grape pomace	2	1.7150	−0.0379	3.4680	81.2	1.3052	1.1425			
Ch/Qu	1	−1.1924	−1.5816	−0.8031	--	--	--			
Gallnut tannic acid	2	0.7042	0.0700	1.3383	71.3	0.1494	0.3866			
***ADG*, g/day**										
Chestnut	5	6.4894	−3.6569	16.6357	98.2	133.1920	11.5409	140.33	7	<0.0001
GMTA	7	−22.5090	−32.1307	−12.8873	96.8	162.8086	12.7596			
Grape extract	1	1.0764	0.6928	1.4601	--	--	--			
Quebracho (Qu)	2	6.0871	4.2437	7.9306	0.0	0	0			
GSP	7	1.6444	0.5049	2.7839	94.7	2.2453	1.4984			
Grape pomace	2	2.6567	−0.3419	5.6553	90.9	4.2610	2.0642			
Ch/Qu	1	−1.1924	−1.5816	−0.8031	--	--	--			
Gallnut tannic acid	2	0.6481	−0.8556	2.1518	94.6	1.1142	1.0556			
***FBW*, kg**										
Chestnut	5	1.7184	−0.0760	3.5128	96.6	4.0292	2.0073	77.54	8	<0.0001
GMTA	3	−0.2500	−0.7892	0.2892	0.0	0	0			
Grape extract	1	1.9873	1.5476	2.4269	--	--	--			
Quebracho	2	0.0868	−0.6090	0.7826	0.0	0	0			
Carob pods	3	0.7726	−1.1539	2.6992	97.6	2.8320	1.6829			
GSP	6	0.9624	−0.1213	2.0461	94.2	1.7380	1.3183			
Grape pomace	2	5.5956	−2.2497	13.4409	95.6	30.6831	5.5392			
Ch/Qu	1	−0.3416	−0.7021	0.0190	--	--	--			
Gallnut tannic acid	2	0.4981	−0.4853	1.4816	88.2	0.4443	0.6666			
***FCR***										
Chestnut	2	−2.8044	−7.0268	1.4179	98.9	9.1831	3.0304	54.46	7	<0.0001
GMTA	7	−1.7433	−3.5702	0.0836	96.6	5.8898	2.4269			
Grape extract	1	−0.4968	−0.8603	−0.1333	--	--	--			
Carob pods	3	0.8295	−0.0430	1.7020	89.7	0.5374	0.7331			
GSP	7	−2.4528	−3.5728	−1.3328	94.3	2.1331	1.4605			
Grape pomace	2	−1.4908	−6.1186	3.1371	96.6	10.7723	3.2821			
Ch/Qu	1	0.8517	0.4775	1.2259	--	--	--			
Gallnut tannic acid	2	−0.0982	−2.6187	2.4223	98.0	3.2399	1.8000			

ADFI: average daily feed intake; ADG: average daily gain; FCR: feed conversion ratio; FBW: final body weight; k: number of studies; SMD: standard mean difference; *I*^2^: Higgens statistics; Q: X^2^ statistics; τ^2^: heterogeneity variance of actual effect size; τ: standard deviation of true effect size: df: degree of freedom.

**Table 3:** Meta-regression of the effects of tannin source dosage on measured growth outcomes.

Variable ^a^	Model Results					Heterogeneity ^b^				
				95% CI ^b^		TM	Mixed Model Effects			
	Estimate	SE ^b^	*p*-Value	Lower	Upper	QM	τ^2^	τ	*I*^2^ (%)	R^2^
***ADFI*, g/day**										
GSP	−0.0118	0.0054	0.0297	−0.0224	−0.0012	4.7277	0.8393	0.9161	90.27	40.74
GMTA	−0.0015	0.0013	0.2556	−0.0040	0.0011	1.2923	2.1981	1.4826	95.53	6.01
***ADG*, g/day**										
Chestnut	−0.0013	0.0046	0.7806	−0.0103	0.0077	0.0776	174.7742	13.2202	99.88	0.00
GMTA	0.0136	0.0102	0.1799	−0.0063	0.0335	1.7983	142.6673	11.9443	96.53	12.37
GSP	−0.0071	0.0090	0.4322	−0.0247	0.0106	0.6169	2.4329	1.5598	95.82	0.00
***FBW*, kg**										
Chestnut	0.0001	0.0008	0.8811	−0.0015	0.0017	0.0224	5.3567	2.3145	97.57	0.00
GSP	−0.0113	0.0158	0.4733	−0.0422	0.0196	0.5143	1.9417	1.3935	95.15	0.00
GMTA	0.0002	0.0007	0.7497	−0.0011	0.0015	0.1018	0.00	0.00	0.00	0.00
Carob pods	−0.0000	0.0001	0.5469	−0.0001	0.0001	0.3628	4.1985	2.0490	98.17	0.00
***FCR***										
GMTA	0.0015	0.0022	0.4800	−0.0027	0.0057	0.4989	6.4586	2.5414	97.33	0.00
GSP	0.0047	0.0091	0.6079	−0.0132	0.0226	0.2633	2.4616	1.5690	95.18	0.00

^a^ ADFI: average daily feed intake; ADG: average daily gain; FBW: final body weight; FCR: feed conversion ratio; GSP: grape seed proanthocyanidins; GMTA: gallnut microencapsulated tannic acid. ^b^ SE: standard error; CI: confidence interval; TM: test of moderators; QM: model sum of squares; *I*^2^: Higgens statistics; τ^2^: heterogeneity variance of true effect size; τ: standard deviation of true effect size; R^2^: percentage of variation explained by the model.

**Table 4:** Meta-regression of the effects of the duration of tannin source supplementation on measured growth outcomes.

Variable ^a^	Model Results					Heterogeneity ^b^				
				95% CI		TM	Mixed Model Effects			
	Estimate	SE	*p*-Value	Lower	Upper	QM	τ^2^	τ	*I*^2^ (%)	R^2^
***ADFI*, g/day**										
GMTA	−0.0113	0.0945	0.9048	−0.1965	0.1739	0.0143	2.8266	1.6813	96.63	0.00
***ADG*, g/day**										
Chestnut	−3.2762	0.2604	<0.0001	−3.7866	−2.7659	158.2981	0.8039	0.8966	83.26	99.40
GMTA	−1.6715	0.1233	<0.0001	−1.9132	−1.4299	183.7752	0.00	0.00	0.00	100.00
***FBW*, kg**										
Chestnut	−1.6715	0.1233	<0.0001	−1.9132	−1.4299	183.7752	0.00	0.00	0.00	100.00
***FCR***										
GMTA	−0.2836	0.0767	0.0002	−0.4339	−0.1333	13.6825	1.7732	1.3316	91.34	69.89

^a^ ADFI: average daily feed intake; ADG: average daily gain; FBW: final body weight; FCR: feed conversion ratio; GMTA: gallnut microencapsulated tannic acid. ^b^ SE: standard error; CI: confidence interval; TM: test of moderators; QM: model sum of squares; *I*^2^: Higgens statistics; τ^2^: heterogeneity variance of true effect size; τ: standard deviation of true effect size; R^2^: percentage of variation explained by the model.

**Table 5:** Meta-regression of the effects of piglets’ age on the association of the tannic source and the measured growth outcomes.

Variable ^a^	Model Results					Heterogeneity ^b^				
				95% CIb		TM	Mixed Model Effects			
	Estimate	SE ^b^	*p*-Value	Lower	Upper	QM	τ2	τ	I2 (%)	R2
***ADFI*, g/day**										
GSP	−0.0650	0.1437	0.6509	0.1437	0.2166	0.2048	1.6443	1.2823	94.85	0.00
***ADG*, g/day**										
GSP	0.0308	0.1837	0.8669	−0.3292	0.3908	0.0281	2.7213	1.6496	96.22	0.00
***FBW*, kg**										
GSP	−0.1648	0.1565	0.2923	−0.4716	0.1419	1.1091	1.7038	1.3053	94.76	1.97
***FCR***										
GSP	−0.1249	0.1722	0.4682	−0.4624	0.2126	0.5263	2.3496	1.5329	94.70	0.00

^a^ ADFI: average daily feed intake; ADG: average daily gain; FBW: final body weight; FCR: feed conversion ratio; GMTA: gallnut microencapsulated tannic acid. ^b^ SE: standard error; CI: confidence interval; TM: test of moderators; QM: model sum of squares; *I*^2^: Higgens statistics; τ^2^: heterogeneity variance of true effect size; τ: standard deviation of true effect size; R^2^: percentage of variation explained by the model.

**Table 6:** Egger’s linear regression test for publication bias.

Items	Bias	SE ^a^	t-Value	df ^a^	*p*-Value
*Growth performance*					
Average daily feed intake	4.1020	3.0431	1.35	23	0.1908
Average daily gain	−2.7391	2.2461	−1.22	25	0.2340
Final body weight	3.3762	2.5073	1.35	23	0.1912
Feed conversion ratio	−9.8262	2.9735	−3.30	23	0.0031
*Serum antioxidant indices*					
MDA	−4.3594	1.1747	−3.71	9	0.0048

^a^ MDA: malondialdehydes; SE: standard error; df: degree of freedom.

**Table 7:** Corrected publication bias of tannin source effect on weaned piglets’ performance and health indices.

Items	df ^b^	Random-Effects Model		Heterogeneity ^b^		
		Effect Size	*p*-Value	Q (*p*-Value)	*I*^2^ (%)	τ^2^
*Growth performance*						
Average daily feed intake	26	0.3874	0.5039	772.74 (<0.0001)	96.6	8.9005
Average daily gain	27	−2.0114	0.4911	1344.43 (<0.0001)	98.0	236.7790
Final body weight	25	0.8494	0.0517	0.0517 (<0.0001)	94.7	4.6938
Feed conversion ratio	30	−0.3920	0.4478	1415.83 (<0.0001)	97.9	8.1086
*Serum antioxidant indices*						
MDA	13	−2.2764	0.5254	181.67 (<0.0001)	92.8	171.0610
T-SOD	11	1.8200	0.0075	125.98 (<0.0001)	91.3	4.7714
CAT	11	1.0790	0.1289	191.54 (<0.0001)	94.3	5.7435
GSH-Px	8	2.6767	0.2590	174.24 (<0.0001)	95.4	47.3510
T-AOC	9	3.2990	0.2666	141.13 (<0.0001)	93.6	82.6910
*Immune indices*						
IgA	4	0.9356	0.8530	485.39 (<0.0001)	99.2	126.7149
IgG	4	5.7818	<0.0001	51.91 (<0.0001)	92.3	9.2207
IgM	5	9.0259	0.6023	448.01 (<0.0001)	98.9	1783.7486

^b^ df: degree of freedom; *I*^2^: Higgens statistics; τ^2^: heterogeneity variance of true effect size; R^2^: percentage of variation explained by the model.

## Data Availability

The datasets used and analyzed during the current study are available from the corresponding author upon reasonable request.

## Supplementary Materials

The following supporting information can be downloaded at: https://www.mdpi.com/article/10.3390/antiox13020236/s1. PRISMA checklist.
